# High GSTP1 inhibits cell proliferation by reducing Akt phosphorylation and is associated with a better prognosis in hepatocellular carcinoma

**DOI:** 10.18632/oncotarget.23420

**Published:** 2017-12-19

**Authors:** Xiaojia Liu, Ning Tan, Hongtao Liao, Guangdong Pan, Qing Xu, Rong Zhu, Liping Zou, Songqing He, Hongguang Zhu

**Affiliations:** ^1^ Department of Pathology, Basic Medical School, Shanghai Medical College, Fudan University, Shanghai 200032, China; ^2^ Laboratory of Liver Injury and Repair Molecular Medicine, Guilin Medical University, Guilin 541001, China; ^3^ Department of Hepatobiliary Surgery, The People’s Hospital of Liuzhou, Liuzhou 545001, China; ^4^ Division of Surgical Pathology, Huashan Hospital, Fudan University, Shanghai 200032, China; ^5^ Department of Hepatobiliary Surgery, Affiliated Hospital of Guangxi Medical University, Nanning 530021, China

**Keywords:** hepatocellular carcinoma, GSTP1, prognosis, cell proliferation, Akt

## Abstract

Glutathione S-transferase (GST) family members promote carcinogenesis and cancer progression. We assessed GST pi 1 (GSTP1) mRNA and protein levels in hepatocellular carcinoma (HCC) using genome databases and tissue microarray (TMA) technology. We found that in cancerous tissues, GSTP1 mRNA was down-regulated in genome databases, and immunohistochemical staining of GSTP1 in 237 HCC cases varied from negative to strongly positive. GSTP1 levels correlated negatively with tumor size and serum alpha-fetoprotein (AFP) in HCC patients, and higher GSTP1 levels associated with longer overall survival (OS) and disease-free survival (DFS). We also found that GSTP1 overexpression restrained HepG2 and Huh7 liver cancer cell proliferation *in vivo* and *in vitro*. GSTP1 arrested the cell cycle at G1/S by up-regulating p21 and p27 and down-regulating p-Akt. Interrupting GSTP1 gene expression promoted liver cancer cell proliferation and increased the percentage of cells in S phase by decreasing levels of p21 and p27 and increasing p-Akt. These results suggest high GSTP1 levels provide a better prognosis through suppression of tumorigenesis in HCC.

## INTRODUCTION

Primary hepatic cancer is the second leading cause of cancer-related mortality worldwide [1]. The most frequently occurring hepatic cancer is hepatocellular carcinoma (HCC), which accounts for 75% of all primary liver cancers and causes more than 600,000 deaths each year [2]. Glutathione S-transferases (GSTs) are isoenzymes that have overlapping substrate specificities and protect cells from cytotoxic and carcinogenic agents [3]. Eight isoforms of cytosolic-soluble GSTs have been recognized in humans, including α, κ, μ, π, σ, θ, ζ, and ω [4]. Glutathione *S*-transferase pi 1 (GSTP1, GenBank accession no. CR450361) has shown both stimulatory [5–7] and inhibitory [8–11] effects on tumorigenesis and cancer prognosis, so we investigated GSTP1’s effect on HCC.

There were three phases to our GSTP1 investigation. In the first phase, we used gene chip data obtained from the Gene Expression Omnibus (GEO, GSE14520-GPL3921) and The Cancer Genome Atlas (TCGA) to analyze GSTP1 mRNA expression in HCC tissues and matched para-tumor tissues. In the second phase, we employed immunohistochemistry (IHC) to determine GSTP1 protein levels in HCC tissues, and analyzed possible correlations to HCC clinicopathological characteristics. We also studied the prognostic impact of GSTP1 with Kaplan-Meier survival curves and Cox regression analyses. In the third phase, we studied functional analysis by altering GSTP1 expression in liver cancer cell lines, and performed *in vitro* and *in vivo* experiments to characterize its biological role in HCC progression.

## RESULTS

### GSTP1 expression level and its association with clinicopathological features in HCC patients

**Figure 1:**
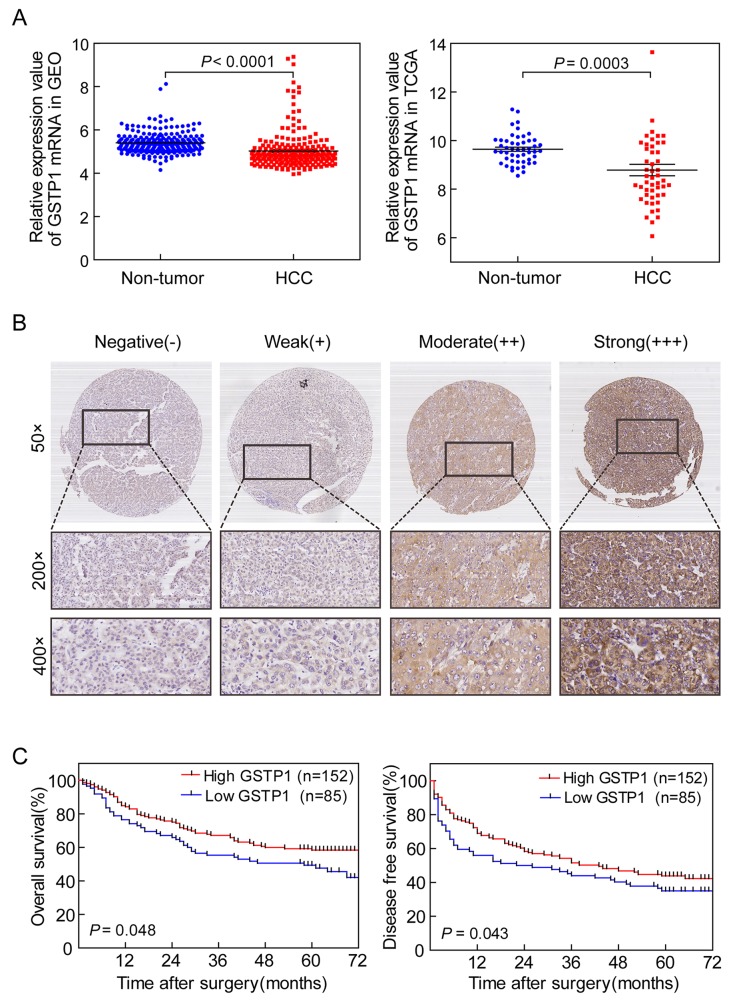
GSTP1 expression in HCC correlated with longer OS and DFS **(A)** Gene chip analysis of GSTP1 mRNA expression in HCC. *Left panel* represented GSTP1 expression in 214 HCC samples from GEO. *Right panel* indicated GSTP1 expression in 50 HCC samples from TCGA. Both databases showed thatGSTP1 mRNA was down-regulated in HCC tissues when compared with adjacent liver tissues **(B)** IHC detection of GSTP1 in HCC. Representative photomicrographs showed negative (−), weak positive (+), moderate positive (++), and strong positive (+++) immunostaining of GSTP1 in HCC specimens (magnification, 50×, 200×, 400×). **(C)** Kaplan-Meier curves of OS and DFS in 237 HCC patients. Patients with lower GSTP1 expression (n=85) had shorter OS and DFS (52 months and 24 months, respectively), while higher GSTP1 (n=152) correlated to longer OS and DFS (62.5 months and 43 months, respectively).

We found that the expression in HCC tissues of GST family members from TCGA was congruent with GEO: GSTA4 is up-regulated, while GSTA1, GSTM1, GSTM2, GSTM5, GSTP1, GSTT1, GSTT2, and GSTZ1 are down-regulated (Supplementary Tables 1 and 2; Supplementary Figures 1 and 2). GSTP1 mRNA was down-regulated in HCC tissues compared with adjacent non-tumor liver tissues (*P* < 0.0001 for GEO and *P* = 0.0003 for TCGA, Figure 1A). IHC results indicated that GSTP1 staining in HCC tissues varied from negative to strong positive (Figure 1B). Negative and weak staining constituted the low GSTP1 group (35.86%), while moderate and strong staining made up high GSTP1 group (64.14%). High GSTP1 was correlated with low serum AFP (*P* = 0.003) and small tumor size (*P* = 0.013, Table 1). However, GSTP1 was not related to HCC patients’ age, gender, hepatitis B surface antigen (HBsAg), liver cirrhosis, Tumor-Node-Metastasis (TNM), portal vein tumor thrombosis (PVTT), or Edmondson-Steiner grade (all *P* > 0.05).

**Table 1:** Correlation between GSTP1 and clinicopathologic features in 237 HCC patients

Variable (missing cases)	Cases	GSTP1				*P*-value
		-	+	++	+++	
**Gender**						0.529
Female	24(10.1%)	1(4.2%)	7(29.1%)	10(41.7%)	6(25%)	
Male	213(89.9%)	25(11.7%)	52(24.5%)	92(43.2%)	44(21.6%)	
**Age**						0.07
≤ 50 ys	112(47.3%)	16(14.2%)	30(26.8%)	46(41.1%)	20(17.9%)	
>50 ys	125(52.7%)	10(8%)	29(23.2%)	56(44.8%)	30(24%)	
**HBsAg (3)**						0.217
Negative	37(15.8%)	4(10.8%)	7(18.9%)	15(40.6%)	11(29.7%)	
Positive	197(84.2%)	22(11.2%)	52(26.4%)	85(43.1%)	38(19.3%)	
**Preoperative serum AFP (2)**						0.003^**^
≤ 400 ng/ml	78(32.8%)	2(2.6%)	20(25.6%)	32(41%)	24(30.8%)	
>400 ng/ml	157(67.2%)	24(15.4%)	39(24.8%)	69(43.9%)	25(15.9%)	
**Tumor Number**						0.639
Single	185(78.1%)	20(10.8%)	47(25.4%)	81(43.8%)	37(20%)	
Multiple	52(21.9%)	6(11.5%)	12(23.1%)	21(40.4%)	13(25%)	
**Tumor size**						0.013^*^
≤ 3 cm	50(21%)	3(6%)	10(20%)	23(46%)	14(28%)	
3-5 cm	59(25%)	5(8.6%)	14(23.7%)	25(42.3%)	15(25.4%)	
> 5 cm	128(54%)	18(14.1%)	35(27.3%)	54(42.2%)	21(16.4%)	
**Edmondson-Steiner grade**						0.475
I	2(0.8%)	0(0%)	0(0%)	1(50%)	1(50%)	
II	17(7.2%)	0(0%)	6(35.3%)	9(52.9%)	2(11.8%)	
III-IV	218(92%)	26(11.9%)	53(24.3%)	92(42.2%)	47(21.6%)	
**PVTT**						0.299
Absent	87(36.7%)	7(8.1%)	19(21.8%)	43(49.4%)	18(20.7%)	
Present	150(63.3%)	19(12.7%)	40(26.7%)	59(39.3%)	32(21.3%)	
**Liver cirrhosis**						0.744
No	80(33.6%)	7(8.8%)	18(22.4%)	41(51.3%)	14(17.5%)	
Yes	157(66.4%)	19(12.1%)	41(26.1%)	61(38.9%)	36(22.9%)	
**TNM**						0.462
I	75(31.5%)	7(9.3%)	15(20%)	39(52%)	14(18.7%)	
II	130(55.1%)	14(10.8%)	36(27.7%)	51(39.2%)	29(22.3%)	
III-IV	32(13.4%)	5(15.6%)	8(25%)	12(37.5%)	7(21.9%)	

* *P*<0.05, ^**^P<0.01, have statistical significance.

**Table 2:** Univariate and multivariate analysis for predictors of OS in 237 HCC patients

Variables	OS			
	Univariate	Multivariate		
	*P*-value	HR	95%CI	*P*-value
Gender (Female vs Male)	0.773	1.423	0.746-2.711	0.284
Age/year (≤ 50 ys vs > 50 ys)	0.361	1.286	0.851-1.944	0.232
HBsAg (Negative vs Positive)	0.651	1.485	0.822-2.684	0.19
AFP (ng/mL) (≤ 400 vs > 400)	0.000^**^	2.178	1.298-3.654	0.003^**^
Number of tumors (Single vs Multiple)	0.000^**^	2.694	1.440-5.038	0.002^**^
Tumor size d/cm (≤ 3 vs 3-5 vs > 5)	0.001^**^	1.889	1.383-2.581	0.000^**^
Edmondson-Steiner grade (I vs II vs III-IV)	0.030^*^	1.167	0.540-2.520	0.694
PVTT (Present vs Absent)	0.047^*^	1.242	0.674-2.291	0.487
TNM (I vs II vs III-IV)	0.000^**^	0.843	0.488-1.454	0.539
GSTP1 (Low vs High)	0.043^*^	0.715	0.510-1.003	0.052

Abbreviations: HR, hazard radio; CI, confidence interval.

^*^P<0.05, ^**^P<0.01, have statistical significance.

### GSTP1 levels and HCC patients’ survival

Kaplan-Meier and log-rank test analyses determined the association between GSTP1 and HCC patients’ survival. In 237 HCC cases with prognostic information, we observed that GSTP1 level was positively associated with OS (Figure 1C *Left*). Patients with lower GSTP1 expression had shorter OS time (median OS = 52 months), while higher GSTP1 suggested longer OS (median OS = 62.5 months). GSTP1 level was also positively associated with DFS (Figure 1C *Right*). Patients with lower GSTP1 levels had a shorter DFS (median OS = 24 months), while higher GSTP1 suggested longer DFS (median DFS = 43 months). The survival curve in the GSTP1 staining groups (−, +, ++, +++) for HCC patients’ OS and DFS showed similar trends (Supplementary Figure 3 and Supplementary Table 3).

**Figure 2:**
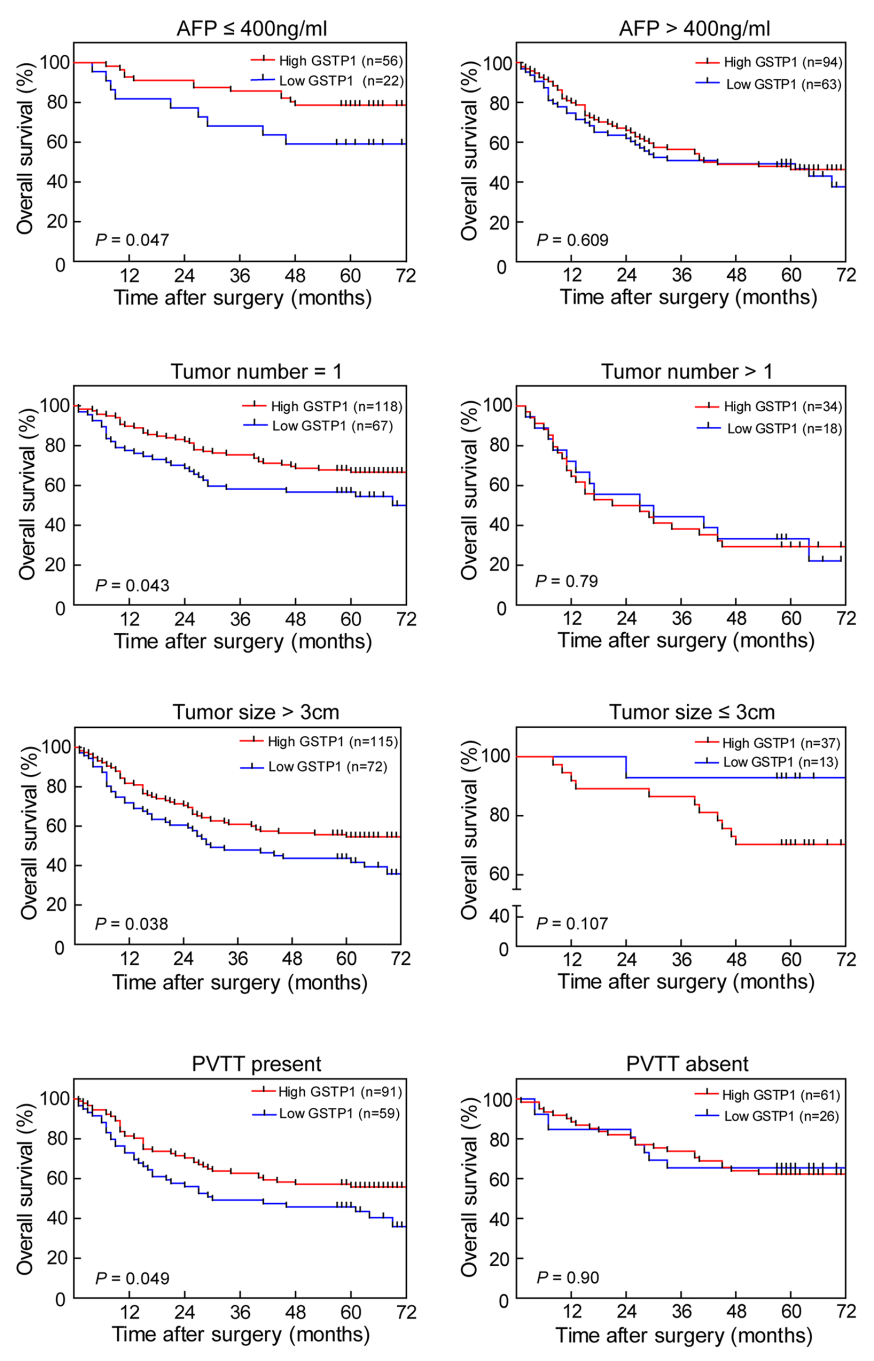
Effect of GSTP1 on OS in HCC subgroups In the AFP ≤ 400ng/ml, single tumor number, tumor diameter > 3 cm, and PVTT-present subgroups, patients with lower GSTP1 expression had shorter OS time (all *P* < 0.05, *left panel*). In the AFP > 400 ng/ml, multiple tumors, tumor diameter ≤ 3 cm, and PVTT-absent subgroups, there was no observable difference between high and low GSTP1 expression on OS (*right panel*).

**Figure 3:**
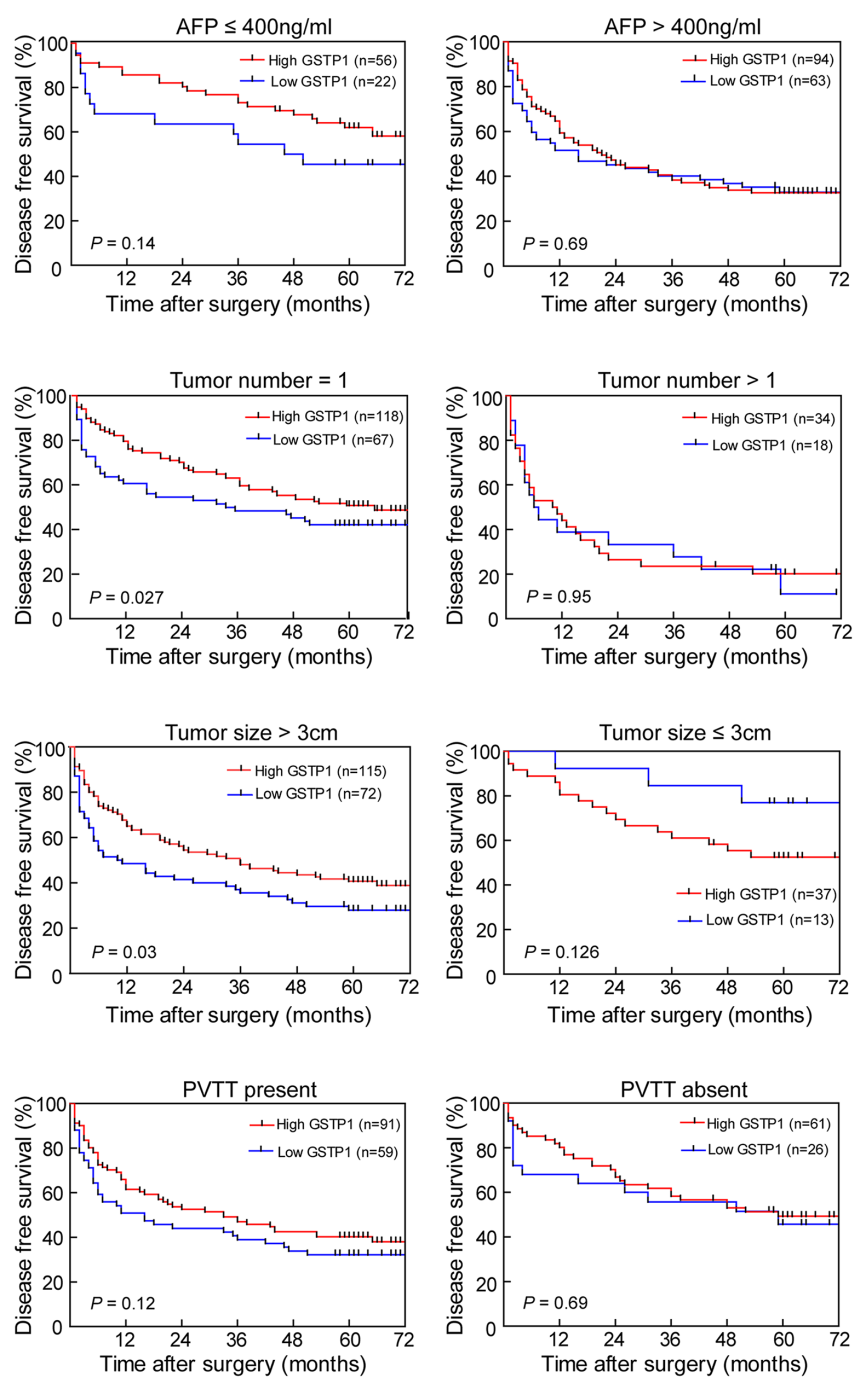
Effect of GSTP1 on DFS in HCC subgroups In the single tumor and tumor diameter > 3 cm subgroups, lower GSTP1 expression indicated shorter DFS (*P* < 0.05, *left panel*). The AFP ≤ 400 ng/ml and PVTT-present subgroups showed the same trend, but it was not significant (*P* = 0.14 and *P* = 0.12, respectively). There was no difference between high GSTP1 and low GSTP1 on DFS in subgroups including AFP > 400 ng/ml, multiple tumors, tumor diameter ≤ 3 cm, and PVTT-absent subgroups (*right panel*).

The prognostic value of GSTP1 was further confirmed by stratified OS and DFS analyses. High GSTP1 expression was correlated with OS (Figure 2) and DFS (Figure 3) in the AFP concentration ≤ 400 ng/ml, single tumor number, tumor diameter > 3cm, and PVTT-present subgroups.

### Univariate and multivariate analysis of prognostic factor in HCC patients

Univariate analysis showed that GSTP1 expression, as well as AFP, tumor number, tumor size, PVTT, TNM stage, and Edmondson-Steiner grade were related to OS (Table 2) and DFS (Table 3) in HCC patients. Multivariate analysis was performed using the Cox Proportional hazards model and the analysis revealed that AFP, tumor number, and tumor size were independent prognostic factors for HCC (all *P* < 0.05), while GSTP1 was not an independent prognostic factor for OS (HR: 0.715, 95% CI: 0.510-1.003, *P* = 0.052) and DFS (HR: 0.859, 95% CI: 0.602-1.226, *P* = 0.403) in HCC patients.

**Table 3:** Univariate and multivariate analysis for predictors of DFS in 237 HCC patients

Variables	DFS			
	Univariate	Multivariate		
	*P*-value	HR	95%CI	*P*-value
Gender (Female vs Male)	0.932	1.493	0.840-2.653	0.172
Age/year (≤ 50 ys vs > 50 ys)	0.206	1.494	1.035-2.156	0.032^*^
HBsAg (Negative vs Positive)	0.306	1.599	0.956-2.674	0.073
AFP (ng/mL) (≤ 400 vs > 400)	0.000^**^	1.898	1.244-2.894	0.003^**^
Number of tumors (Single vs Multiple)	0.000^**^	2.086	1.116-3.900	0.021^*^
Tumor size d/cm (≤ 3 vs 3-5 vs > 5)	0.002^**^	1.538	1.196-1.978	0.001^**^
Edmondson-Steiner grade (I vs II vs III-IV)	0.033^*^	1.601	0.806-3.182	0.179
PVTT (Present vs Absent)	0.034^*^	0.911	0.515-1.613	0.750
TNM (I vs II vs III-IV)	0.000^**^	1.064	0.611-1.854	0.827
GSTP1 (Low vs High)	0.049^*^	0.859	0.602-1.226	0.403

Abbreviations: HR, hazard radio; CI, confidence interval.

^*^P<0.05, ^**^P<0.01, have statistical significance.

### Effect of GSTP1 overexpression on hepatic cancer cell proliferation *in vitro* and *in vivo*

**Figure 4:**
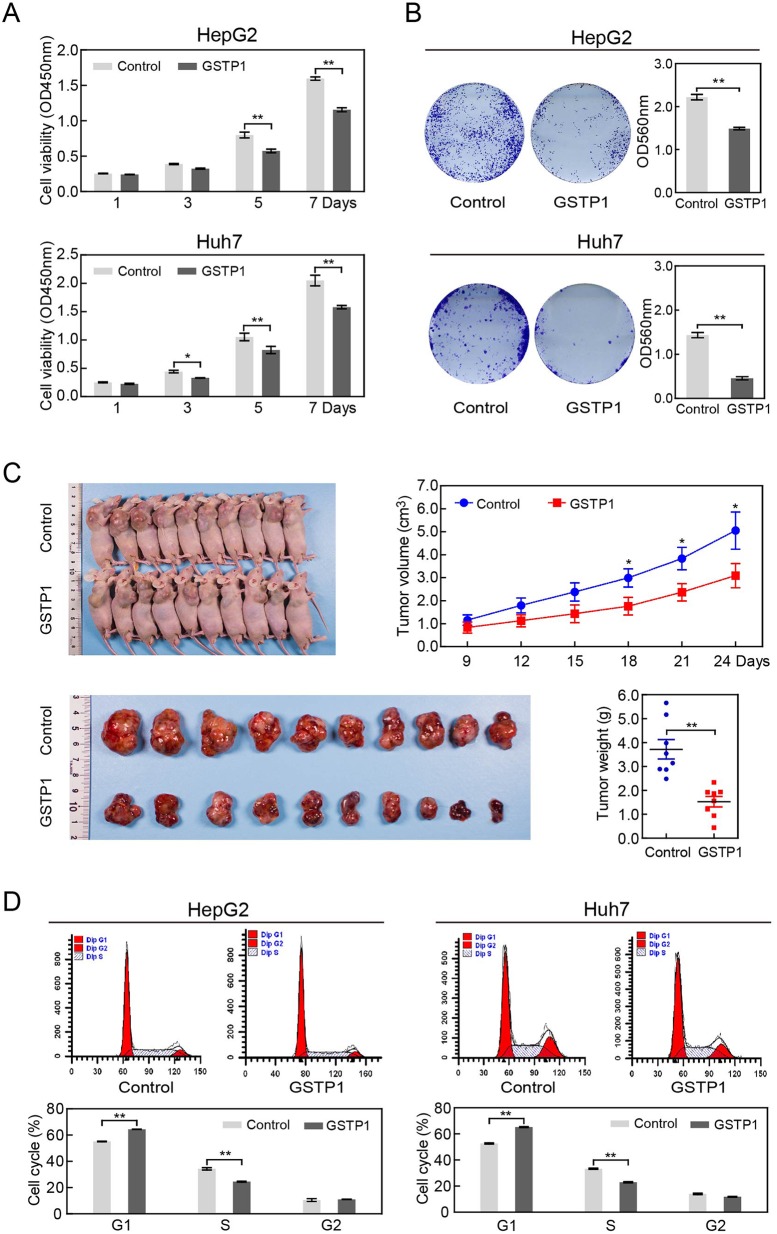
GSTP1 overexpression decreased liver cancer cell proliferation *in vitro* and *in vivo* **(A)** CCK8 assay showed that GSTP1-transfected liver cancer cells had decreased cell viability than in control. **(B)** Colony size and density in GSTP1 overexpression groups were smaller and rarer than that in control groups. **(C)** In the xenograft assay, tumor volume and weight were lower in mice over-expressing GSTP1. **(D)** When compared to control groups, GSTP1 overexpression led to an accumulation of cells in G1 phase and a decrease of cells in S phase. Data were mean ± SD of three biological replicates (^*^P < 0.05, ^**^P < 0.01).

GSTP1 protein levels in a group of liver cancer cell lines and normal liver cell lines were shown in Supplementary Figure 4. HepG2 (without GSTP1) and Huh7 (with GSTP1) were chosen as experimental cells. The effect of GSTP1 overexpression on liver cancer cell proliferation was measured by a Cell Counting Kit-8 (CCK8) assay. The optical density at 450 nm (OD450) of HepG2 in the control group was 0.249 ± 0.001, 0.355 ± 0.013, 0.834 ± 0.079, and 1.695 ± 0.103 at 1, 3, 5, and 7 days, respectively (Figure 4A). GSTP1 overexpression reduced the viability of HepG2 cells in a time-dependent manner, with OD450 of 0.236 ± 0.002 (Day 1), 0.299 ± 0.014 (Day 3), 0.477 ± 0.037 (Day 5), and 0.902 ± 0.151 (Day 7). A similar trend occurred in Huh7 cells.

Colony-formation assays were used to evaluate the long-term effect of GSTP1 on cell survival. GSTP1 overexpression led to a decrease on cell colony formation ability in both HepG2 and Huh7 cells (Figure 4B). We investigated whether GSTP1 could prevent Huh7 progression *in vivo*. Nude mice were randomly divided into two groups (10 mice per group), and subcutaneously injected in the right flank with Huh7-GSTP1 cells or Huh7-control cells. Tumors from the GSTP1 overexpression group grew slower than the control group (Figure 4C). The tumor volumes of GSTP1-transfected mice at 18 days, 21 days, and 24 days were reduced compared with those in the control group (*P* < 0.05). Tumor mass was lower in the GSTP1 overexpression group at the time of harvest (*P* < 0.01). Furthermore, flow cytometric (FCM) assays indicated that in both HepG2 and Huh7 cells, GSTP1 overexpression led to an accumulation of cells in G1 phase and a decrease in S phase compared with the control groups (all *P* < 0.05, Figure 4D).

### Effect of GSTP1 shRNA on hepatic cancer cell proliferation

**Figure 5:**
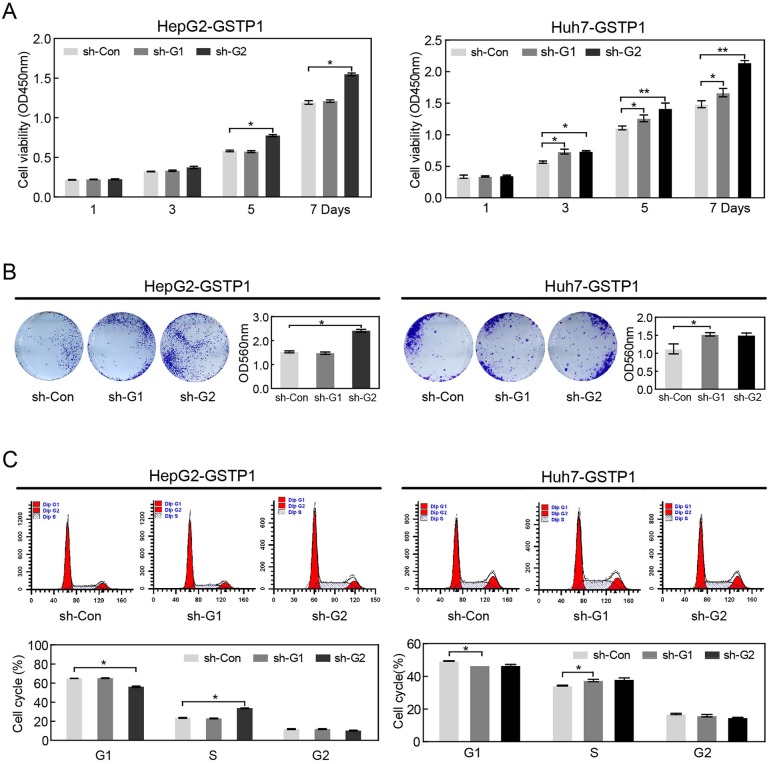
GSTP1 shRNA enhanced the proliferation ability of liver cancer cells *in vitro* **(A)** CCK8 assay showed that HepG2 and Huh7 cell proliferation increased in day 5 and day 7 after GSTP1 shRNA (sh-G2) transfection. **(B)** GSTP1 shRNA groups (sh-G2) formed more cell colonies than sh-control groups. **(C)** FCM showed that GSTP1 shRNA (sh-G2) led to a decrease in G1 phase but an increase in S phase. Data were mean ± SD of three biological replicates (^*^P < 0.05, ^**^P < 0.01).

We used shRNA with GSTP1-specific target sequences (sh-G1 and sh-G2) to silence GSTP1 in HepG2 and Huh7. CCK8 assay showed that HepG2 and Huh7 proliferation in shG2 groups was increased in day 5 and day 7 after transfection (all *P* < 0.05, Figure 5A). The colony formation ability of HepG2 and Huh7 cells increased in GSTP1 shRNA groups (sh-G2) compared with control groups (Figure 5B). Cell lysis concentration was measured by an automatic microplate reader and the OD560 values of HepG2-GSTP1 sh-Con and HepG2-GSTP1 sh-G1 (1.612 ± 0.013 and 1.586 ± 0.038, respectively) were lower than the OD560 of sh-G2 (2.561 ± 0.027). Huh7 cells showed the same trend. FCM results also showed that there was a decrease in G1 phase but an increase in S phase in GSTP1 shRNA groups (sh-G2) (Figure 5C).

### GSTP1 modulated the expression of cell cycle proteins

**Figure 6:**
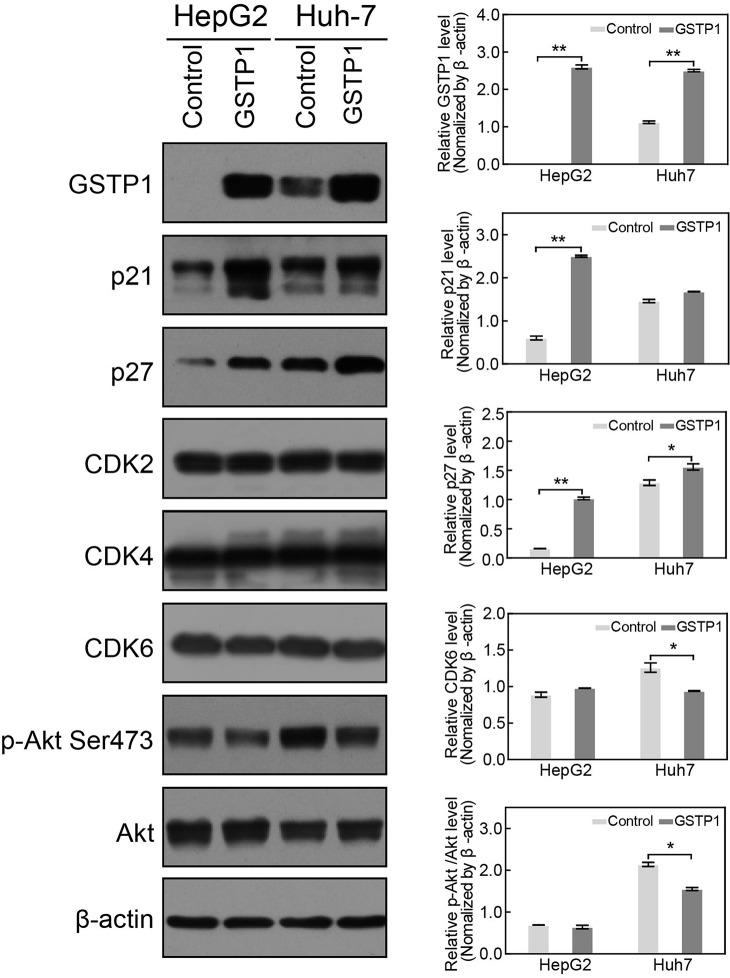
GSTP1 overexpression up-regulated p21 and p27, but down-regulated p-Akt Western blot showed that GSTP1 protein was overexpressed in GSTP1-transfected liver cancer cells, indicating a successful transfection. In HepG2 and Huh7 cells, GSTP1 overexpression increased p21 and p27 protein expression and moderately decreased CDK6 (with no statistically significant), while CDK2 and CDK4 levels did not change. While the total Akt amount remained unchanged, western blotting results indicated that p-Akt Ser473 levels decreased in cells overexpressing GSTP1 compared to controls. Data were mean ± SD of three replicates (^*^P < 0.05, ^**^P < 0.01).

**Figure 7:**
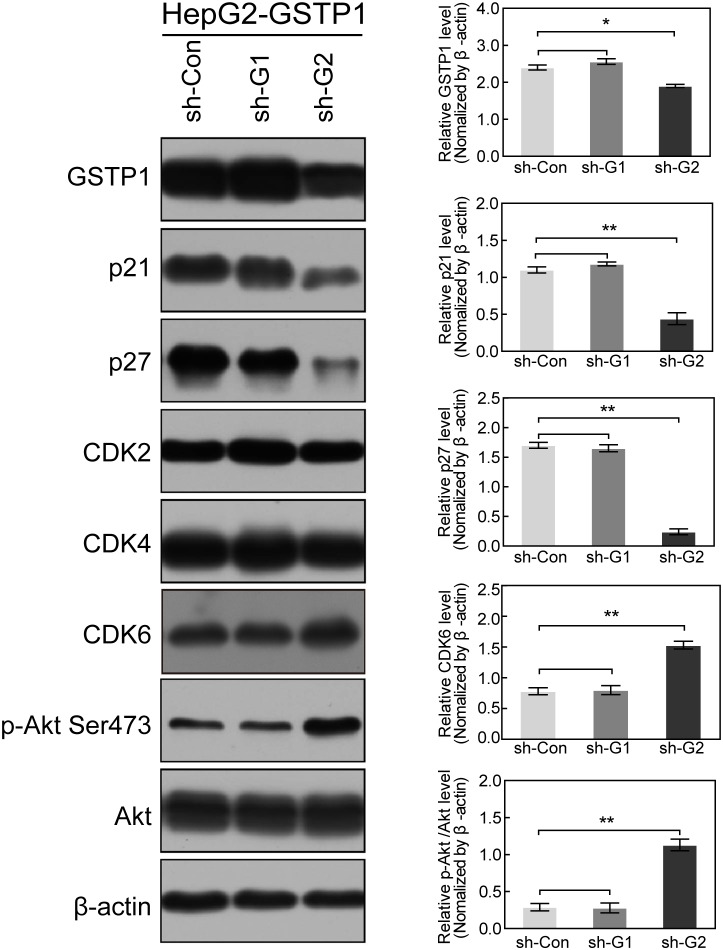
GSTP1 shRNA down-regulated p21 and p27, but up-regulated p-Akt and CDK6 ShRNA decreased GSTP1 expression in HepG2-GSTP1 cells, and a better transfection effect was achieved in the sh-G2 group. Down-regulation of GSTP1 decreased p21 and p27 protein expression, but increased CDK6 and p-Akt Ser473 expression in HepG2 cells. The total amount of Akt remained unchanged. Data were mean ± SD of three replicates (^*^P < 0.05, ^**^P < 0.01).

To explore the mechanism of GSTP1-induced cell proliferation and cell cycle alteration in HCC cells, we extracted total protein from GSTP1-ov/sh groups and control groups. In GSTP1-overexpressing groups of HepG2 and Huh7, western blot showed a mild CDK6 decrease when compared with the control group (though it was not significant, Figure 6). GSTP1 overexpression did not affect CDK2 or CDK4, but it did decrease p-Akt Ser473 activation and elevate p21 and p27 protein levels. After suppressing GSTP1 expression with shRNA, we found p-Akt Ser473 and CDK6 were up-regulated while p21 and p27 were down-regulated (Figure 7).

## DISCUSSION

Previous GSTP1 studies have reached contradicting conclusions, so we investigated GSTP1’s effects on tumor progression and prognosis in HCC. We began our study by exploring genetic databases and conducting gene chip analyses. According to both the Gene Expression Omnibus and The Cancer Genome Atlas, only GSTA4 mRNA levels were higher in tumor tissues than non-tumor tissues. Gene chip analysis showed that the levels of GSTP1 and seven other GSTs were lower in HCC tissues than adjacent non-tumor liver tissues. These results align with those of Zhang et al. [12], who found decreased GSTP1 activity more often in HCC tissue than para-tumor tissue.

We observed a negative correlation between GSTP1 expression and both tumor size and serum AFP. This relationship could suggest a positive correlation between GSTP1 expression and better HCC outcomes. Smaller tumor size has been reported during the early stage of tumorigenesis and tumor volume can increase concomitantly with tumor progression in most cases [13]. AFP is elevated in the serum of patients with hepatic lesions, and acts as an HCC biomarker. While the mechanism of the negative association between GSTP1 and AFP in HCC patients is not defined, Zhu M et al. found that AFP could promote liver cancer stem cell initiation by activating the PI3K/Akt signal pathway [14]. We found that GSTP1 could decrease p-Akt in liver cancer cell lines, and speculated that GSTP1 may inhibit AFP expression.

Higher GSTP1 levels in tumor tissues indicated a better OS and DFS for HCC patients enrolled in our study. The median OS was 52 months for low GSTP1 patients, and 62.5 months for high GSTP1 patients. The median DFS survival was 29 months for low GSTP1 groups, and 45 months for patients with high GSTP1. Although multivariate analysis showed that GSTP1 could not be regarded as an independent risk factor for predicting HCC prognosis, univariate analysis revealed low GSTP1 expression was associated with shorter OS and DFS. This association suggests GSTP1 can act as a protective factor.

The clinical significance of GSTP1 suggested that GSTP1 might also influence the biological behavior of liver cancer cells. Kou [15] showed that GSTP1 overexpression could reduce the survival of HepG2 cells and induce cell cycle arrest by suppressing the transcriptional activity of Stat3. However, Tao N et al. found that low GSTP1 could activate JNK-mediated signaling pathways and abolish apoptosis in HCC cells [16]. In our research, we found that GSTP1 expression inhibited HepG2 and Huh7 proliferation and colony formation. We also found that GSTP1 could prevent Huh7 progression in nude mice. Given that GSTP1 suppresses hepatic cancer cell growth *in vitro* and *in vivo*, we further confirmed that GSTP1 was a protective factor.

An uncontrolled cell cycle and malignant cell proliferation are the main characteristics of cancer. The eukaryotic cell cycle is controlled by a regulatory network [17], which proceeds through tightly regulated transitions. The transition points during the cell cycle can be grouped into three main waves, namely G1-to-S, G2-to-M, and M-to-G1. An uncontrolled G1/S transition allows cells to progress into S phase in an unrestrained fashion, which is a hallmark of cancer [18]. In GSTP1 overexpression groups, our flow cytometry results showed that the cell population accumulated in G1, but declined in S phase; in GSTP1-silenced groups, cell population was higher in the S phase than in G1. Thus, GSTP1’s inhibition on cancer progression may be accomplished by arresting the cell cycle at the G1/S transition in HCC cells.

Cell cycle progression is regulated by cyclins, cyclin-dependent kinases (CDKs), and cyclin-dependent kinases inhibitors (CKI) [19]. To explore the mechanism of the GSTP1-induced cell cycle arrest at G1/S transition in HCC, intracellular levels of cell cycle modulators were determined by western blotting. In the GSTP1-overexpressing HepG2 and Huh7 cells, we found that p27 and p21 levels were increased, while CDK6 was decreased. GSTP1 shRNA could down-regulate p21 and p27, but up-regulate CDK6. CDK6 could form a complex with Cyclin D and regulate cell progression through the G1 phase of the cell cycle, and expresses high in various cancers [20, 21]. The p27 protein could inhibit CyclinD-CDK4/CDK6 activity by interfering with CDK4/CDK6 in the activation segment [22, 23]. Just like p27, p21 is well positioned to function as both a sensor and an effector of multiple anti-proliferative signals [24]. Our data suggested that up-regulation of p21 and p27 might inhibit CDK6 activation, leading to the G1 arrest and suppressed growth in GSTP1-overexpressing HCC cells.

We found that GSTP1 overexpression decreased p-Akt Ser473, and GSTP1 shRNA could increase p-Akt Ser473, without any effect on the expression of total Akt. We concluded that p-Akt can regulate p21 and p27 directly or indirectly [25, 26] in liver cancer cells with different GSTP1 levels.

In summary, GSTP1 mRNA was down-regulated in tumor compared with para-tumor liver tissues in HCC. HCC patients with lower AFP and smaller tumor size were more likely to have higher GSTP1, which was correlated to better OS and DFS. We found that GSTP1 overexpression could inhibit liver cancer cell proliferation *in vivo* and *in vitro* by arresting cell cycle progression at the G1/S transition. This could be related with the up-regulation of p21 and p27 protein, and down-regulation of CDK6 and p-Akt. All these results indicate GSTP1 may be applied as potential prognostic biomarker and new therapeutic target in HCC.

## MATERIALS AND METHODS

### Ethics statement

This investigation was conducted in accordance with the Declaration of Helsinki and national and international guidelines approved by the Institutional Review Board of Guilin Medical University. We obtained written informed consent from all patients. Animal care and experiments were performed in strict accordance with the “Guide for the Care and Use of Laboratory Animals” and the “Principles for the Utilization and Care of Vertebrate Animals,” and were approved by the Experimental Animal Ethical Committee of Guilin Medical University.

### Online database data retrieval

Gene expression data for GSTs in HCC, including paired tumors and adjacent non-tumor liver tissues, were identified from NCBI GEO (GSE14520-GPL3921, http://www.ncbi.nlm.nih.gov/geo/) and TCGA (https://tcga-data.nci.nih.gov/tcga/). This study met the publication guidelines provided by GEO and TCGA.

### Patient selection

A total of 237 HCC patients who underwent hepatectomies between January 2006 and July 2010 were randomly selected from Guilin Medical University Affiliated Hospital in this retrospective study. The criteria for case selection were as follows: (1) pathological diagnosis of hepatocellular carcinoma, (2) none of the patients received anti-cancer therapies prior to surgery, and (3) no history of other cancer. Tumor stage was defined according to the American Joint Committee on Cancer (AJCC, 2010, 7th edition) Tumor-Node-Metastasis (TNM) staging system. Tumor grade was assigned by the Edmondson-Steiner grading system.

All the patients were being regularly followed for up to 90 months, with a median survival time of 50.5 months (range, 1-90 months). Patients were monitored by abdomen ultrasonography, chest X-ray, and a test for the serum AFP concentration every month during the first year after surgery, and every 3 months thereafter. The overall survival (OS) was defined as the length of time between the surgery and death, or the last follow-up examination. The disease-free survival (DFS) was calculated from the date of tumor resection until the detection of tumor recurrence.

### TMA and immunohistochemistry

Hematoxylin and eosin (H&E)-stained slides from all patients were reviewed and identified by two experienced pathologists, and the representative cores were premarked in the paraffin blocks. Tissue cylinders with a diameter of 1.0 mm were punched from the marked areas of each donor block and incorporated into a recipient paraffin block.

Sections of 4 μm thickness were placed on slides coated with 3-aminopropyltriethoxysilane and dried at 60°C for 2 hours. Paraffin sections were deparaffinized in xylene, and rehydrated with decreasing concentrations of ethanol (100%, 95%, 90%, and 85%, for 5 minutes each). Antigens were unmasked by microwave irradiation for 5 minutes in pH 6.0 citric buffer. Endogenous peroxidase activity was blocked by incubating the slides in 3% hydrogen peroxide/phosphate-buffered saline, and non-specific binding sites were blocked with 10% goat serum. The slides were incubated with the mouse monoclonal primary antibody against GSTP1 (mAb #3369, 1:800 dilution, cytoplasmic staining; CST, USA) overnight at 4°C in a moist chamber, and then conjugated with secondary antibody labeled with horseradish peroxidase (mAb #7076, 1:1000 dilution; CST, USA) for 60 minutes at room temperature. Finally, the slides were stained for 30 seconds using the DAB Kit (Boster Bio-Engineering Company, Wuhan, China) according to the manufacturer’s instructions. The slides were then counterstained with hematoxylin, differentiated by hydrochloric alcohol, dehydrated in ascending series of ethanol, cleared in xylene, sealed with neutral balsam, and scanned by Olympus virtual slides workshop120 (VS120, Olympus, USA).

### Evaluation of staining

DAB staining regions for GSTP1 were scored by 2 pathologists blinded to the clinical parameters. The score standard for the staining intensity was as follows: 0 (negative), 1 (weak), 2 (moderate), 3 (strong). The score of staining extent was 0 (10%), 1 (11%-25%), 2 (26%-50%), 3 (51%-75%), and 4 (76%-100%). The final GSTP1 expression score was calculated with the intensity score + extent score, ranging from 0 to 7. The staining results were divided into 4 categories based on the sum of scores: 0-1 was negative (−), 2-3 was weak positive (+), 4-5 was moderate positive (++), and 6-7 was strong positive (+++). A final score ≤ 3 was defined as low expression, and > 3 as high expression.

### Cell culture and transfection

Human liver cancer cell line HepG2 was purchased from the American Type Culture Collection (ATCC, Manassas, VA, USA), and Huh7 was obtained from Shanghai Institute of Cell Biology, Chinese Academy of Sciences. HepG2 and Huh7 were routinely cultured in Dulbecco’s Modified Eagle Medium (DMEM, Cat No. 8113262, Gibco, US), supplemented with 10% fetal bovine serum (FBS, Lot.1438121, Gibco, US), penicillin (100 U/ml, Cat No.15140-122, Invitrogen, US), and streptomycin (100 mg/ml, Cat No.15140-122, Invitrogen, US), at 37°C in a 5% humidified CO2 incubator. The lentivirus vectors for up-regulation/down-regulation of GSTP1 were obtained from GenePharma Co., Ltd (Shanghai, China). Liver cancer cell lines were infected with the lentivirus vectors constitutively expressing GSTP1 or specific GSTP1 shRNA by Lipofectamine® 2000 Transfection Reagent, according to the procedures of the manufacturer (Invitrogen). Transfection efficacy was confirmed by western blot.

### Cell proliferation assay

The effect of GSTP1 on liver cancer cell proliferation was detected using CCK8 assay. Cells in the logarithmic phase of growth were seeded in 96-well plates (1 × 10^3^/well) and cultured for 24, 72, 120, and 168 hours. Subsequently, 10μL of CCK-8 solution (Dojindo Laboratories, Kumamoto, Japan) were added into each well and incubated for 2 hours. Optical density was measured at a wavelength of 450 nm by an automatic microplate reader (Bio Tek, USA). Triplicate wells were assayed for each experiment, and three independent experiments were performed. Data were expressed as the OD450 mean ± S.D.

### Colony formation assay

GSTP1 overexpression/shRNA groups and their control groups were placed in six-well plates at 2000 cells per well in triplicate. Cells were incubated in medium supplemented with 10% FBS at 37°C for 2 weeks to let the viable cells propagate to sizable colonies. Colonies were fixed by 4% paraformaldehyde for 15 min, stained in a 0.1% crystal violet and 20% methanol dye solution for 5 min, then photographed. The cells were lysed with 1% glacial acetic acid solution (GAAS, Cat No. 537020, Sigma-Aldrich, US), and the lysate was transferred into a new 96-well plate. Lysate concentrations of each well were quantified at an absorbance of 560 nm using an automatic microplate reader (Bio Tek, USA). Data were expressed as the OD560 mean ± S.D.

### Cell cycle analysis

Cells were harvested and a single cell suspension was adjusted to 1 × 10^6^ cells/ml, then fixed in ice-cold 70% ethanol overnight. The fixed cells were washed twice with phosphate-buffered saline and stained with a freshly-prepared solution containing 25 μg/ml propidium iodide (PI, Cat No. P4170, Sigma, US), 10 μg/ml RNase A (Cat No. R5125, Sigma, US) in phosphate-buffered saline (PBS) for 30 min in the absence of light. Each sample was analyzed using BD FACS caliber cytometry. Three independent experiments were performed for each assay. The results were presented as percentages of the total cell count in different phases of the cell cycle, namely the G1 phase, S phase, and G2 phase.

### Tumor xenograft in nude mice

Male athymic nude mice were obtained from SLAC Laboratory Animal Co., Ltd (Shanghai, China) at 5 weeks of age and kept under controlled conditions (12h light and dark cycles) with free access to food and water in SPF animal laboratory. We generated xenograft tumors using Huh7 cells stably overexpressing GSTP1 or their controls. These cancer cells were suspended in PBS and Matrigel (1:1 ratio), and 3×10^6^ cells were subcutaneously injected into the right flanks of each mouse. Matrigel was used to improve the attachment and differentiation of both GSTP1 stably over-expressed cells and control cells in nude mice. Ten mice were used in each group, and subcutaneous tumor volume was monitored for 24 days following injection. Tumor growth was monitored by measuring the tumor size every three days with a digital caliper. The tumor volume was calculated using the following formula: length (mm) × width^2^ (mm^2^) × 0.5. At the endpoint, the mice were sacrificed, necropsies were performed, and the xenografts were measured. The Mann-Whitney U test was performed to compare the variables in tumor growth rate between the two groups of the nude mice.

### Western blot assay

Protein samples were extracted using RIPA lysis buffer containing a protease inhibitor cocktail and a phosphatase inhibitor cocktail (Roche, Rotkreuz, Switzerland), and then separated with SDS-PAGE and transferred onto nitrocellulose membranes (Bio-Rad, Hercules, USA). The membranes were blocked with 5% non-fat milk in Tris-buffered saline (TBS) containing 0.1% Tween-20 for 1.5 hours at room temperature. The blots were probed with the relevant primary antibodies overnight at 4°C, and probed with a horseradish peroxidase-conjugated secondary antibody for 1 hour. An enhanced chemiluminescence detection method (Pierce ECL Western Blotting Substrate, Thermol, USA) was used to visualize the blots. All primary antibodies were purchased from CST company, including anti-GSTP1 (#3369), anti-p-Akt Ser473 (#4060), anti-Akt (#9272), anti-p21 (#2947), anti-p27 (#3686), anti-CDK2 (#2546), anti-CDK4 (#12790), and anti-CDK6 (#13331). Anti-β-actin (#3700) was used as the internal control antibody.

### Statistical analysis

All experiments were repeated at least three times. Data were analyzed with IBM SPSS software version 20.0, and expressed as mean ± SD. A value of *P* < 0.05 was considered significant. GSTP1 levels between HCC tumor and pari-tumor tissues in TCGA and GEO databases were analyzed by Wilcoxon signed-rank test. Kruskal–Wallis one-way analysis of variance (ANOVA) or Spearman rank correlation analysis was performed to determine the relevance between GSTP1 and clinicopathological variables of HCC patients. Kaplan-Meier and log-rank test analyses were performed to determine the association between GSTP1 levels and HCC patient survival. Multivariate analyses were performed by multivariate Cox proportional hazard regression model to assess the prognostic variables in HCC patients. Mann-Whitney U test or Student’s test was performed to compare the variables of two groups in CCK8 assay, colony formation assay, flow cytometry, tumor xenograft assay, and western blot.

## SUPPLEMENTARY MATERIALS FIGURES AND TABLES



## Abbreviations

Alpha-fetoprotein: AFP
American Joint Committee on Cancer: AJCC
American Type Culture Collection: ATCC
Analysis of variance: ANOVA
Cell Counting Kit-8: CCK8
Confidence interval: CI
Cyclin-dependent kinases: CDK
Cyclin-dependent kinases inhibitors: CKI
Disease-free survival: DFS
Dulbecco’s Modified Eagle Medium: DMEM
Fetal bovine serum: FBS
Flow cytometric: FCM
Glacial acetic acid solution: GAAS
Gene Expression Omnibus: GEO
Glutathione S-transferases: GSTs
Glutathione S-transferase pi 1: GSTP1
Hazard radio: HR
Hematoxylin and eosin: H&E
Hepatitis B surface antigen: HBsAg
Hepatocellular carcinoma: HCC
Immunohistochemical: IHC
Optical density 450nm: OD450
Overall survival: OS
phosphate-buffered saline: PBS
Portal vein tumor thrombosis: PVTT
Propidium iodide: PI
shRNA: Small hairpin ribonucleic acid
The Cancer Genome Atlas: TCGA
Tissue microarray: TMA
Tris-buffered saline: TBS
Tumor-Node-Metastasis: TNM.
