# Supplementary material for: High GSTP1 inhibits cell proliferation by reducing Akt phosphorylation and is associated with a better prognosis in hepatocellular carcinoma
Source: Oncotarget. 2017 Dec 19;9(10):8957–71. doi: 10.18632/oncotarget.23420 (PMC5823662; doi:10.18632/oncotarget.23420)
Supplement: Supplementary file 1 [file oncotarget-09-8957-s001.pdf]

High GSTP1 inhibits cell proliferation by reducing Akt phosphorylation and is associated with a better prognosis in hepatocellular carcinoma

SUPPLEMENTARY MATERIALS

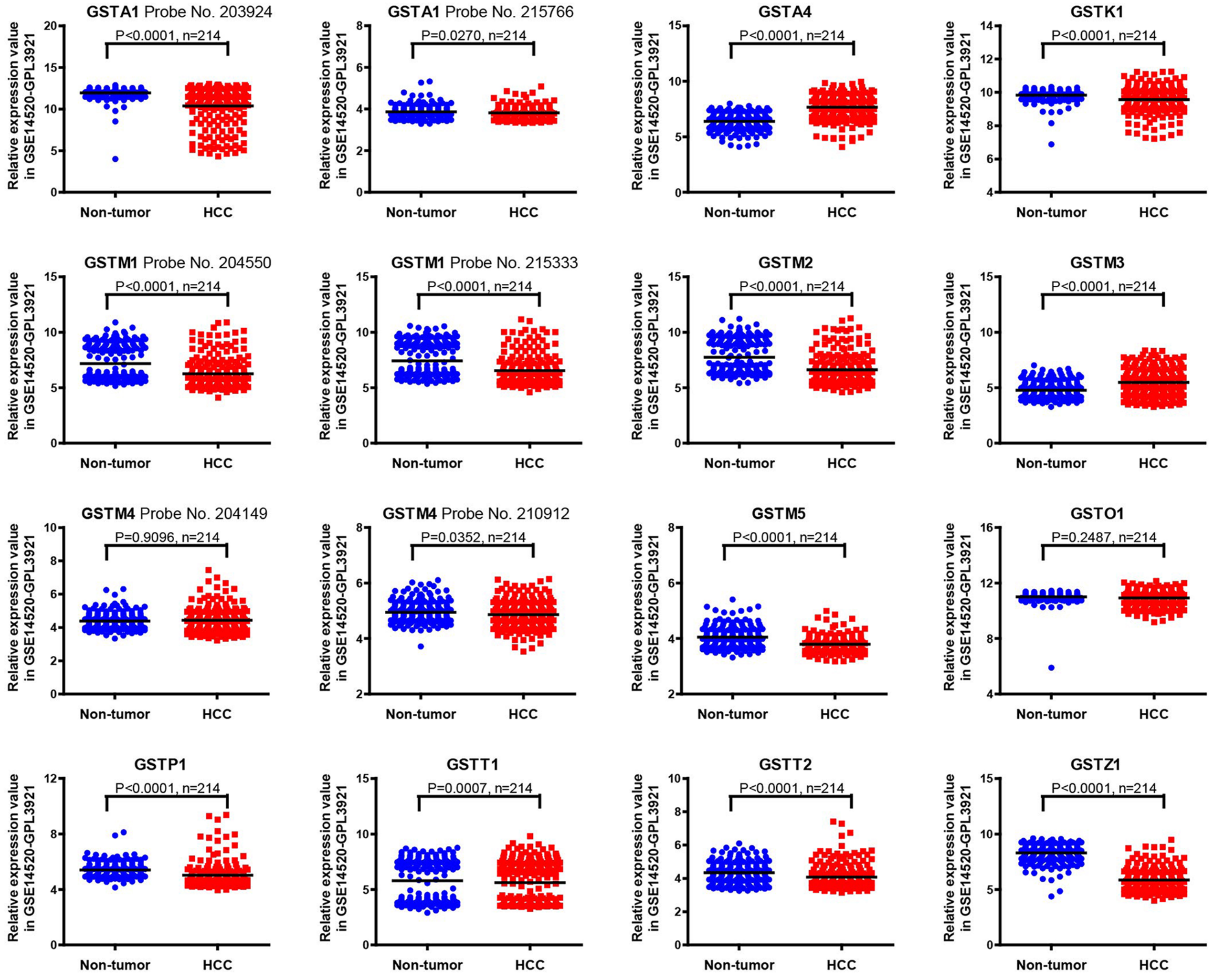

Supplementary Figure 1: GSTs expression in HCC patients in GEO. In GEO, GSTA4 and GSTM3 mRNA in HCC tumor tissues were significantly higher than that in para-tumor liver tissues. While GSTA1, GSTK1, GSTM1, GSTM2, GSTM5, GSTP1, GSTT1, GSTT2 and GSTZ1 mRNA were lower in HCC tissues than that in para-tumor liver tissues (n=214, all P<0.05).

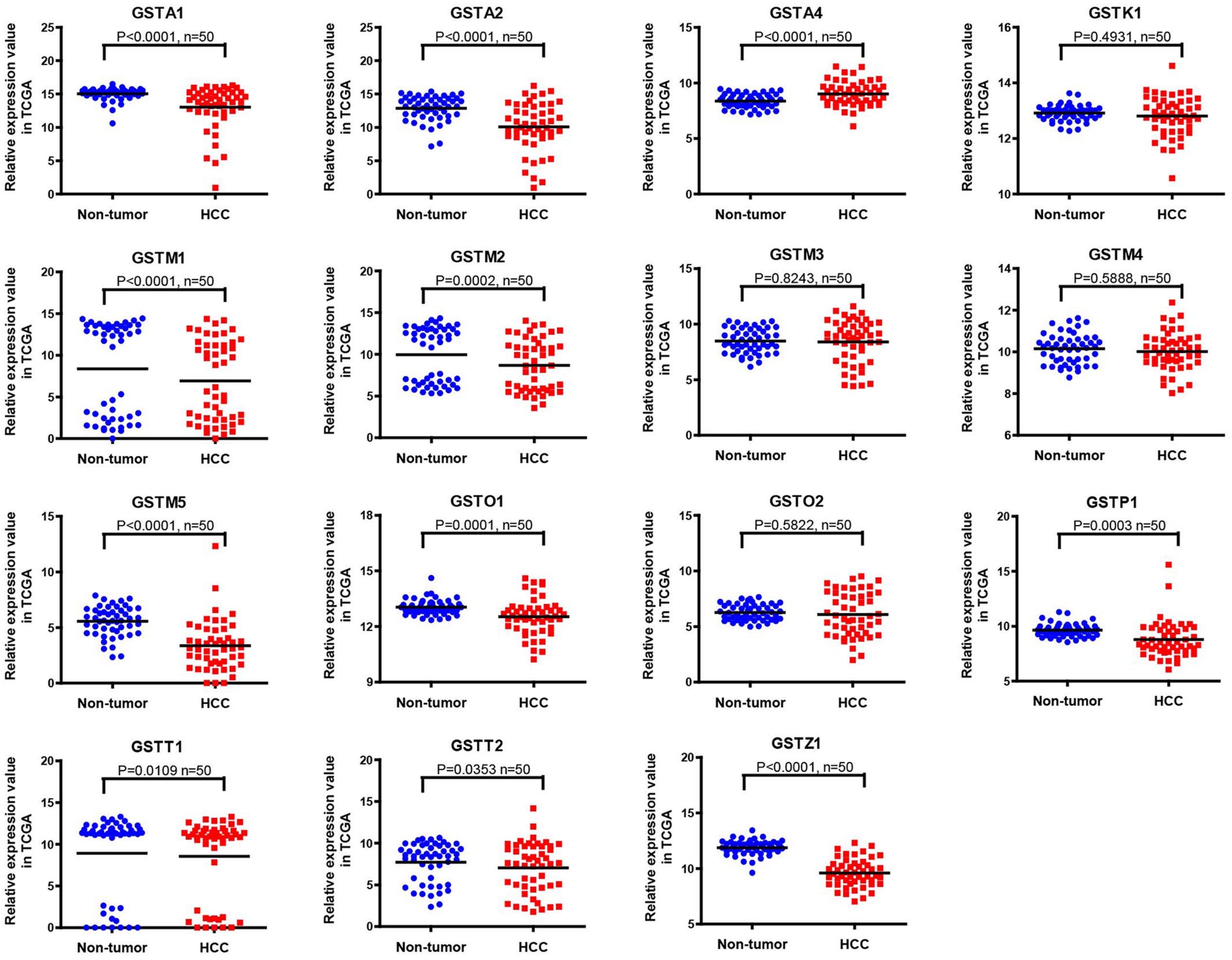

Supplementary Figure 2: GSTs expression in HCC patients in TCGA. In TCGA, the mRNA expression of GSTA4 in HCC tumor tissues was higher than that in para-tumor liver tissues. While GSTA1, GSTA2, GSTM1, GSTM2, GSTM5, GSTO1, GSTP1, GSTT1, GSTT2 and GSTZ1 mRNA were lower in HCC tissues than that in para-tumor liver tissues (n=50, all $P<0.05$).

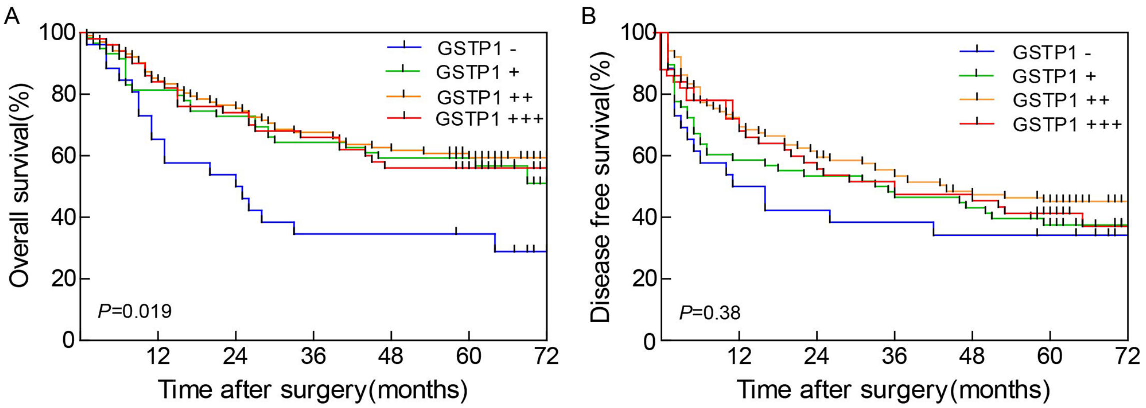

Supplementary Figure 3: OS and DFS for HCC patients with different GSTP1. (A) GSTP1(-) group had a much lower OS when compared with GSTP1(+), (++) and (+++) groups. **(B)** GSTP1(-) had a lower DFS (only 13.5 months), while GSTP1(+), (++) and (+++) patients' DFS were higher than 30 months.

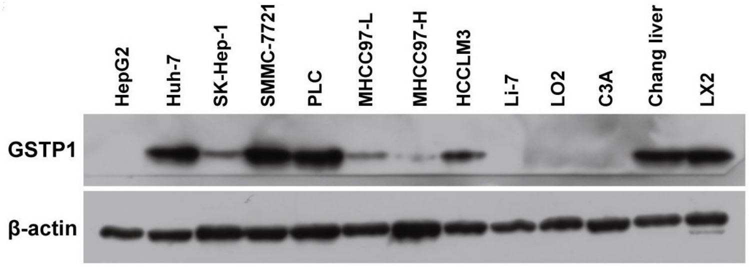

Supplementary Figure 4: Protein level of GSTP1 in a groups of liver cancer cell lines and normal liver cell lines checked by western blot. GSTP1 was not expressed in liver cancer cells including HepG2, Li7, C3A and normal liver cell line LO2. There was a very low GSTP1 in liver cancer cells including SK-Hep1, MHCC97-L, MHCC97-H and HCCLM3. There was a certain amount of GSTP1 in liver cancer cells (Huh7, SMMC-7721 and PLC), hepatic stellate cell LX2 and normal liver cell line Chang liver.

Supplementary Table 1: Expression of GSTs family in human HCC tissues and matched non-tumor liver tissues detected by Gene-chip Human Genome U133A Array (GEO)

		Mean± Std. Deviation	Std. Error	Minimum	Median	Maximum	Lower 95%CI	Upper 95% CI	*P*-value
GSTA1-203924	Non-Tumor	11.93±0.72	0.05	3.99	12.04	12.87	11.83	12.02	<0.0001**
	HCC	10.34±2.27	0.16	4.32	11.15	13.03	10.06	10.67	
GSTA1-215766	Non-Tumor	3.87±0.29	0.02	3.29	3.85	5.33	3.83	3.91	0.027*
	HCC	3.81±0.29	0.02	3.32	3.78	5.09	3.77	3.85	
GSTA4	Non-Tumor	6.40±0.74	0.05	4.09	6.43	7.98	6.30	6.49	<0.0001**
	HCC	7.66±1.11	0.08	4.07	7.78	8.99	7.51	7.81	
GSTK1	Non-Tumor	9.83±0.34	0.02	6.88	9.89	10.33	9.79	9.88	<0.0001**
	HCC	9.57±0.78	0.05	7.21	9.67	11.24	9.46	9.67	
GSTM1-204550	Non-Tumor	7.17±1.54	0.11	5.16	6.24	10.88	6.97	7.38	<0.0001**
	HCC	6.26±1.38	0.10	4.11	5.69	10.88	6.67	6.45	
GSTM1-215333	Non-Tumor	7.41±1.51	0.10	5.34	6.60	10.58	7.21	7.61	<0.0001**
	HCC	6.53±1.31	0.09	4.59	6.05	11.16	6.35	6.71	
GSTM2	Non-Tumor	7.74±1.47	0.10	5.41	7.00	11.20	7.54	7.94	<0.0001**
	HCC	6.62±1.39	0.09	4.59	6.12	11.26	6.43	6.81	
GSTM3	Non-Tumor	4.76±0.74	0.05	3.28	4.62	7.01	4.66	4.86	<0.0001**
	HCC	5.47±1.25	0.09	3.27	5.33	8.36	5.30	5.64	
GSTM4-204149	Non-Tumor	4.39±0.46	0.03	3.34	4.28	6.31	4.32	4.45	0.9096
	HCC	4.43±0.73	0.05	3.23	4.30	7.45	4.33	4.53	
GSTM4-210912	Non-Tumor	4.95±0.38	0.03	3.71	4.87	6.10	4.89	4.99	0.0352*
	HCC	4.86±0.50	0.03	3.53	4.82	6.14	4.79	4.93	
GSTM5	Non-Tumor	4.05±0.37	0.03	3.32	4.00	5.41	4.00	4.09	<0.0001**
	HCC	3.80±0.28	0.02	3.18	3.77	4.99	3.76	3.84	
GSTO1	Non-Tumor	11.00±0.40	0.03	5.90	11.04	11.46	10.95	11.06	0.249
	HCC	10.93±0.61	0.04	9.18	11.01	12.15	10.85	11.01	
GSTP1	Non-Tumor	5.40±0.50	0.03	4.15	5.32	8.12	5.34	5.47	<0.0001**
	HCC	5.03±0.88	0.06	3.94	4.81	9.37	4.91	5.15	
GSTT1	Non-Tumor	5.78±1.82	0.13	2.90	6.41	8.76	5.54	6.03	0.0007**
	HCC	5.63±1.83	0.13	3.30	5.62	9.80	5.38	5.87	
GSTT2	Non-Tumor	4.34±0.61	0.04	3.26	4.29	6.10	4.26	4.42	<0.0001**
	HCC	4.08±0.73	0.05	3.14	3.79	7.41	3.98	4.18	
GSTZ1	Non-Tumor	8.29±0.81	0.06	4.37	8.45	9.61	8.19	8.41	<0.0001**
	HCC	5.85±1.02	0.07	4.00	5.62	9.47	5.71	5.98	

*P<0. 05, **P<0.01, have statistics significant.
Abbreviations: GSTs, Glutathione S-transferase.

Supplementary Table 2: Expression of GSTs family in human HCC tissues and matched non-tumor liver tissues detected by TCGA

		Mean± Std. Deviation	Std. Error	Minimum	Median	Maximum	Lower 95%CI	Upper 95% CI	*P*-value
GSTA1	Non-Tumor	15.03± 0.96	0.135	10.60	15.25	16.50	14.76	15.30	<0.0001**
	HCC	13.02± 3.30	0.467	0.94	14.01	16.29	12.08	13.96	
GSTA2	Non-Tumor	12.86± 1.85	0.262	7.146	13.15	15.38	12.34	13.39	<0.0001**
	HCC	10.08± 3.65	0.516	0.942	10.20	16.23	9.05	11.12	
GSTA4	Non-Tumor	8.63± 0.63	0.09	7.15	8.44	9.45	8.18	8.54	<0.0001**
	HCC	9.01± 1.10	0.16	6.09	8.95	11.47	8.70	9.33	
GSTK1	Non-Tumor	12.91± 0.28	0.039	12.27	12.92	13.63	12.83	12.99	0.493
	HCC	12.81± 0.71	0.099	10.57	12.82	14.61	12.61	13.01	
GSTM1	Non-Tumor	8.38± 5.56	0.79	0	12.02	14.41	6.79	9.95	<0.0001**
	HCC	6.92± 4.79	0.68	0	5.91	14.35	5.55	8.28	
GSTM2	Non-Tumor	9.95± 3.29	0.47	5.32	11.60	14.32	9.01	10.89	0.0002**
	HCC	8.68± 3.07	0.43	3.57	8.63	14.02	7.81	9.55	
GSTM3	Non-Tumor	8.48± 1.10	0.16	6.16	8.26	10.28	8.17	8.79	0.82
	HCC	8.41± 1.94	0.28	4.43	8.84	11.60	7.85	8.96	
GSTM4	Non-Tumor	10.15± 0.72	0.10	8.77	10.22	11.62	9.96	10.36	0.59
	HCC	10.02± 0.92	0.13	8.03	10.00	12.37	9.76	10.28	
GSTM5	Non-Tumor	5.56± 1.33	0.19	2.34	5.62	7.88	5.18	5.93	<0.0001**
	HCC	3.36± 2.25	0.32	0	3.12	12.32	2.73	4.00	
GSTO1	Non-Tumor	13.04± 0.42	0.06	12.35	13.01	14.62	12.92	13.16	<0.0001**
	HCC	12.53± 0.97	0.14	10.23	12.54	14.60	12.26	12.81	
GSTO2	Non-Tumor	6.27± 0.72	0.10	4.98	6.21	7.64	6.07	6.48	0.58
	HCC	6.08± 1.96	0.28	1.99	5.80	9.51	6.48	6.64	
GSTP1	Non-Tumor	9.64± 0.60	0.09	8.56	9.60	11.29	9.47	9.82	0.0003**
	HCC	8.79± 1.62	0.23	6.07	8.41	15.60	8.32	9.26	
GSTT1	Non-Tumor	8.91± 4.89	0.69	0	11.33	13.29	7.52	10.31	0.01*
	HCC	8.56± 4.81	0.68	0	10.92	13.30	7.19	9.93	
GSTT2	Non-Tumor	7.72± 2.34	0.33	2.36	8.44	10.64	7.055	8.39	0.035*
	HCC	7.03± 2.99	0.42	1.78	7.57	14.17	6.18	7.88	
GSTZ1	Non-Tumor	11.88± 0.66	0.09	9.62	11.91	13.43	11.69	12.07	<0.0001**
	HCC	9.60± 1.25	0.18	7.03	9.43	12.30	9.24	9.95	

**P*<0. 05, ***P*<0.01, have statistics significant.
Abbreviations: GSTs, Glutathione S-transferase.

Supplementary Table 3: OS and DFS for 237 HCC patients with different GSTP1

GSTP1	Cases	Median Survival for OS (months)	Median Survival for DFS (months)
-	27	24.5	13.5
+	58	>72	34
++	102	>72	44
+++	50	>72	36
